# Glucocorticosteroids and ciclosporin do not significantly impact canine cutaneous microbiota

**DOI:** 10.1186/s12917-018-1370-y

**Published:** 2018-02-23

**Authors:** Giovanni Widmer, Lluís Ferrer, Claude Favrot, Judy Paps, Kevin Huynh, Thierry Olivry

**Affiliations:** 1 Department of Infectious Diseases and Global Health, Cummings School of Veterinary Medicine at Tufts University, 200 Westboro Road, North Grafton, MA USA; 2 Department of Clinical Sciences, Cummings School of Veterinary Medicine at Tufts University, 200 Westboro Road, North Grafton, MA USA; 3 Clinic for Small Animal Internal Medicine, Vetsuisse Faculty, University of Zürich, Winterthurerstrasse 260, -8057 Zürich, CH Switzerland; 4 Department of Clinical Sciences, College of Veterinary Medicine, North Carolina State University, 1060 William Moore Drive, Raleigh, NC 27607 USA

**Keywords:** Prednisone, Ciclosporin, 16S amplicon sequencing, Microbiota, *Malassezia*, *Papillomavirus*, Principal coordinates analysis

## Abstract

**Background:**

As prednisone and ciclosporin can have immunosuppressive effects and have been considered potential predisposing factors for skin infections, we investigated the impact of these drugs on the diversity of the cutaneous microbiota, the abundance of *Malassezia* and infection with *Papillomaviruses.*

**Results:**

Six atopic, asymptomatic Maltese-beagle dogs were treated with ciclosporin for one month and then with prednisone for another month, with a one-month wash-out between treatments. The dogs were sampled on the abdomen and pinna before and after each treatment using a swab. Samples for *Papillomavirus* detection were obtained with cytobrush sticks. The bacterial microbiota was characterized using 16S amplicon high-throughput sequencing. *Malassezia* populations were quantified with nested real-time PCR targeting the ribosomal internal transcribed spacer 1. The diversity and composition of cutaneous microbiota was not impacted in a detectable manner by any of the treatments. As observed for the bacterial microbiota, *Malassezia* populations were not affected by treatment. Three dogs were positive for *Papillomavirus* at more than one timepoint, but an association with treatment was not apparent.

**Conclusions:**

Ciclosporin and prednisone at doses used for the treatment of atopic dermatitis do not impact the canine cutaneous microbiota in a detectable manner.

**Electronic supplementary material:**

The online version of this article (10.1186/s12917-018-1370-y) contains supplementary material, which is available to authorized users.

## Background

Cutaneous infections are very prevalent in atopic dogs; in most cases, they are caused by microorganisms that are considered normal inhabitants of the canine skin, such as *Staphylococcus pseudintermedius* or *Malassezia pachydermatis* [1]. The mechanisms that trigger the proliferation of a commensal cutaneous organism and leads to a bona fide infection are poorly understood. As skin infections are thought to worsen the clinical manifestations of atopic dermatitis, understanding the mechanisms underlying their development is of high interest.

Both glucocorticoids and ciclosporin are extensively used to treat atopic dermatitis in dogs. These drugs are recommended as first line therapies in the recently updated guidelines for the management of this disease [2]. As both drugs, depending on their dose, can have immunosuppressive effects, some authors have suggested that their prolonged use could favor the development of skin infections. For instance, treatment with glucocorticoids is considered to predispose to the development of superficial pyoderma in dogs, and relapsing urinary tract infections are recognized as a common side effect of glucocorticoid treatment in this species [1, 3, 4].

There is less evidence for ciclosporin predisposing to skin infections, but an increased rate of urinary tract infections in atopic dogs treated with this drug has been reported [5]. Finally, the development of viral papillomas in dogs has been mentioned in different studies as one on the side effects of ciclosporin therapy, probably due to an alteration of in the immune mechanism controlling viral replication [6, 7].

One way to assess if ciclosporin and glucocorticoids predispose to the development of skin infections is to analyze the impact of such treatments on the skin microbiota using high-throughput sequencing. These techniques enable the evaluation of the relative abundance of cutaneous bacteria and diversity of the skin microbiota [8, 9]. In this approach, changes in the microbiome could be used as proxy to assess the risk for cutaneous infections. In support of this assertion, skin infections were recently shown to be preceded by a diminished bacterial microbiota diversity in human and canine atopic patients [10, 11].

To examine the impact of ciclosporin and prednisone on the canine skin microbiota, we designed a longitudinal study with dosages used to treat canine allergic skin diseases. We found that these treatments have no discernable impact on the diversity of canine bacterial microbiota nor on *Malassezia* or *Papillomavirus* populations of the skin.

## Methods

### Dogs and treatments

Six co-housed Maltese-beagle crossbred atopic dogs numbered 1 to 6 were used for this study. The dogs are purpose-bred for allergy research. There were three intact males and three females aged between 3 and 8 years (average: 5.7 years). These dogs had been sensitized in preceding years to *Dermatophagoides farinae* house dust mites [12]. For this study, allergen challenges and lesion induction were not performed, and the dogs did not exhibit skin lesions at any time. At first, six dogs were treated orally with 5 mg/kg of ciclosporin once daily for one month. After a 1-month wash out, the dogs were treated with 0.75 mg/kg of prednisone per os once daily for one month. The interventions were approved by the Institutional Animal Care and Use Committee at NC State University. Drugs used in this study were those commercially available. They were purchased from this university’s Veterinary Hospital Pharmacy. No anesthesia was used and the dogs were not euthanized upon conclusion of the in vivo study.

### Sample collection

Samples were collected aseptically at six timepoints over a period of 7 months. These timepoints are numbered T0 (pre-treatment) - T5, where T0 samples were collected 19 days before initiation of ciclosporin treatment, T1 on the day ciclosporin was first administered, T2 at conclusion of ciclosporin treatment, T3 44 days after the end of ciclosporin treatment and upon initiation of prednisone treatment, T4 at the end of the prednisone treatment and T5 after a 30-day washout period. Samples were collected in all cases from two sites: ventral pinna and inguinal skin. For the bacterial microbiota and for the *Malassezia* analyses, a sterile swab was used (Isohelix, Harrietsham, Kent, UK). Swabs were rubbed for 15 s on each side of the swab within an area of approximately 4 cm^2^. For the evaluation of *Papillomavirus*, the samples were obtained using sterile cytobrush sticks (CytoSmear, Purfybr, Munster, Indiana) [13]. Samples were stored at 4 °C for no more than 72 h before extraction and final storage at − 80 °C.

### 16S rRNA amplicon sequencing for bacterial microbiota characterization

DNA was extracted from swabs using the Power Soil DNA isolation kit (MoBio, Carlsbad, CA). Bacterial populations were characterized using 16S ribosomal RNA (rRNA) amplicon high-throughput sequencing. A portion of the bacterial 16S rRNA ribosomal rRNA gene from the V1 V2 variable region was amplified using primers 27F and 338R [14], generating an amplicon of > 300 nucleotides (nt). A first PCR of 20 cycles using primer 27F (AGAGTTTGATYMTGGCTCAG) and 338R (TGCTGCCTCCCGTAGGAGT) was followed by a second PCR of 20 cycles to incorporate Illumina adapters and a unique 6-nt barcode. All PCR experiments included a positive and negative control reaction. Barcoded PCR products were electrophoresed on 2.5% agarose gel to check for the presence of amplification product of the correct size. The concentration of amplicon DNA was quantified by measuring absorbance at 260 and 280 nm. Amplicons were pooled at approximately equal concentration and the pooled library sequenced at the Tufts University Genomics core facility (tucf.org) using an Illumina MiSeq sequencer and a custom sequencing primer [15]. Amplicons were sequenced single-end, 300 nt. An average of 92,567 (standard deviation (SD) = 72,127) sequence reads were obtained per barcode. FASTQ files were deposited in the European Nucleotide Archive under accession number PRJEB15158. To assess the level of experimental noise in the sequence data, we duplicated three samples (Additional file 1). Duplicates were amplified separately from the same DNA sample and each amplicon tagged with a unique 6-nt barcode.

### Bioinformatics and statistical analysis

Bioinformatics analysis was performed using mothur essentially as described [16–18]. Briefly, random subsamples of 5000 sequences were aligned using Clustal Omega [19] and curated to remove sequencing errors. Chimeras were removed using Uchime [20] as implemented in mothur. Sequences were denoised using program pre.cluster [18] using a difference threshold of 2 nt. Differences between bacterial populations (β diversity) were quantified using the weighted Unifrac phylogenetic distance [21]. Unifrac is a metric of pairwise phylogenetic dissimilarity frequently used in microbial ecology. It is a measure of the fraction of branches in a phylogenetic tree which are unique to either of the two populations being compared. Matrices of pairwise distance between 89 samples were computed in *mothur* and visualized on Principal Coordinate Analysis (PCoA) plots using GenAlEx [22]. Sequences were classified using the Naïve Bayesian classifier [23] with template and taxonomy reference files downloaded from the Ribosomal Database Project [24]. The minimum bootstrap value for taxonomic assigment was set at 70%.

To measure α diversity, we used the Shannon and Berger-Parker indices. The latter index is equal to the proportion of the most abundant Operational Taxonomic Unit (OTU), where an OTU is a collection of similar sequences and can be viewed as a proxy of species. In contrast to Shannon diversity, increasing Berger-Parker diversity indicates decreasing diversity.

The significance of clustering of samples based on Unifrac distance was tested using ANOSIM [16]. Variation Partitioning Analysis [25] as implemented in CANOCO [26] was used to assess the relative contribution of two independent variables, dog and time, to the microbiome profile.

### *Malassezia* nested PCR

A nested PCR protocol was developed and used to amplify a 142-nt fragment of the *Malassezia* Internal Transcribed Spacer (ITS) 1. The following primers targeting the ribosomal repeat sequence of *M. pachydermatis* were used; outside primer forward: AGGTTTCCGTAGGTGACCT, outside primer reverse: TTCGCTGCGTTCTTCATCGA [27]. The inside, nested, forward primer (unpublished) was AACCCGTGTGCACTT, and the reverse nested primer CGTTGTCGAAAGTTG. To quantify *Malassezia* DNA, amplification curves were generated with a RT PCR assay on a Roche LightCycler instrument. PCR experiments were controlled with positive and negative controls and specificity of the amplification confirmed by sequencing a representative amplicon. As a positive control, a sample from a dog with severe *Malassezia* overgrowth (20–25 organisms/high power field) was used. The number of PCR cycles needed to reach the instrument’s detection threshold, known as the Crossing Point (C_t_), was used as a measure of relative *Malassezia* DNA concentration. A standard curve was calculated to convert C_t_ values to *Malassezia* DNA copy number. Seven 10-fold serial dilutions of a *Malassezia* DNA reference sample with an average of 41.7 cells per high-power field were prepared and PCR amplified using the nested primer sets described above. A first order linear regression with the equation

$$ \mathsf{Ct}=-{\mathsf{4.06}}^{\ast }\ \mathsf{\log}\left(\mathsf{copy}\ \mathsf{number}\right)+\mathsf{43.94} $$

and r^2^ = 0.998 was obtained and used to convert PCR C_t_ values to ITS1 copy number.

### *Papillomavirus* PCR

Samples collected with a cytobrush were kept at 4 °C for no longer than 24 h and stored at − 18 °C until extraction. To pellet cells and debris, tubes (still containing the cytobrush tip) were centrifuged at 15000 *g* for 10 min. The cytobrush tip was removed and the saline supernatant aspirated leaving 25 μl in the tube. DNA was extracted using the DNeasy extraction kit (Qiagen, Hilden, Germany) following the manufacturer’s instructions. DNA was eluted in 100 μl sterile water. RT PCR was used to identify and quantify *Papillomavirus* DNA in the samples. The PCR amplified a region of the L1 gene (canPVf/FAP64; CTTCCTGAWCCTAAYMAKTTTGC/ CCWATATCWVHCATNTCNCCATC). This probe detects all viruses belonging to the genus *Papillomavirus*. The papillomavirus PCR was performed at the Veterinary School of the University of Zurich, Switzerland, as described elsewhere [13].

## Results

### Analysis of bacterial microbiota

#### Impact of treatments

**Fig. 1:**
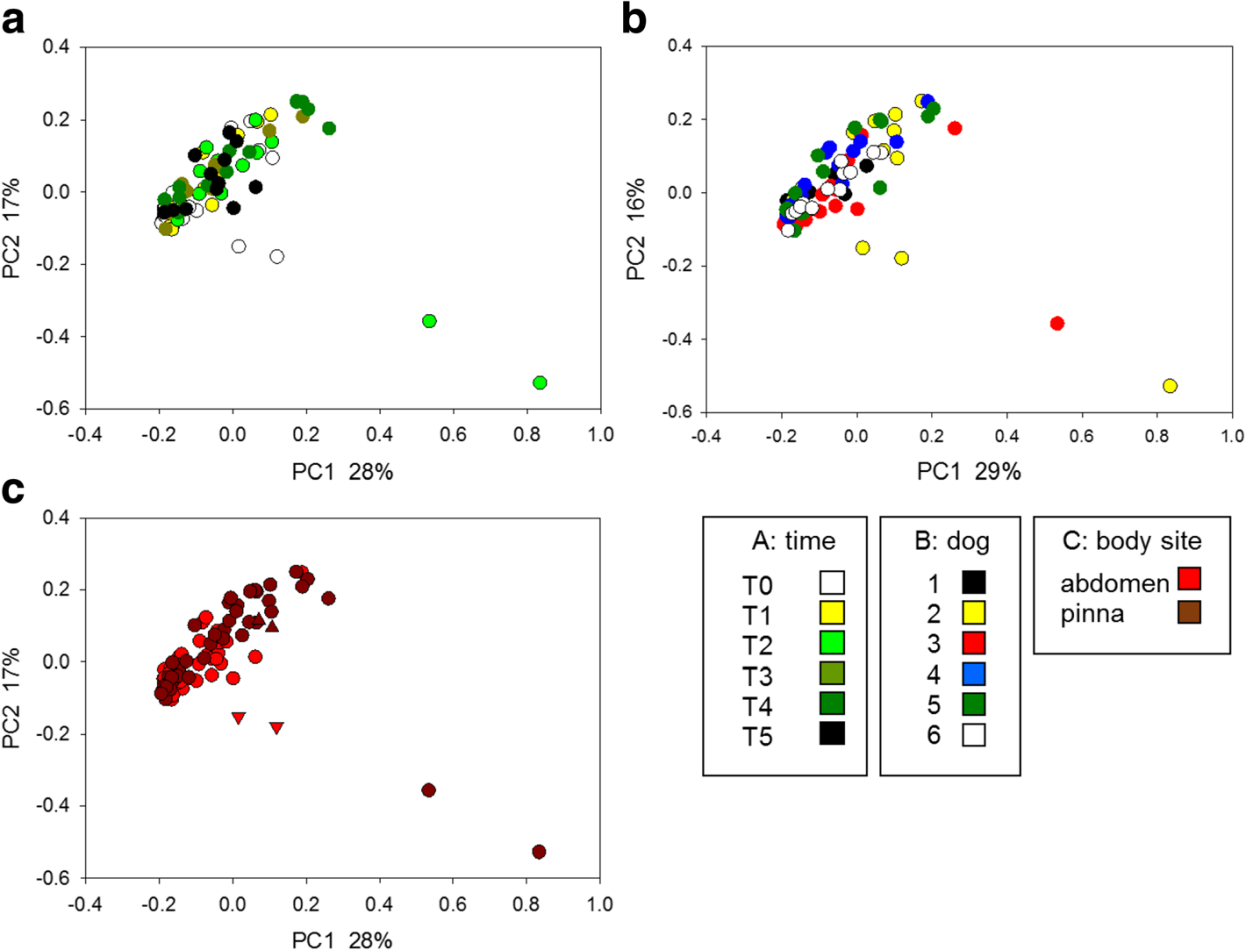
Principal Coordinates Analysis of 89 swab samples. Colors represent time of collection (**a**), dog (**b**) and body site (**c**) as shown in the key. The complete list of samples is shown in supplementary material. Duplicated samples extracted from the same sample and barcoded separately are indicated with matching triangles in plot C (see also Additional file 1)

We used PCoA of pairwise weighted Unifrac distance matrices to display differences in 16S rRNA sequence profile in different dog skin swabs. Figure 1 shows three versions of the same PCoA of 89 samples. The plots are colored according to date of collection (A), dog (B) and body site (C). There is no visible indication that ciclosporin or prednisone treatment, initiated at timepoint T1 and T3, respectively, affected the skin bacterial microbiota (panel A). Statistical analysis of weighted Unifrac distances between samples collected at the six timepoints confirmed this interpretation (Table 1). T1 and T2 samples, and T3 and T4 samples were not significantly different (*p* = 0.262 and *p* = 0.345, respectively). Only two comparisons, T0 versus T2 and T0 versus T5 were significantly different. Since these timepoints do not bracket the treatments, we suspected a temporal effect unrelated to treatment and concluded that the tested interventions had not discernable effect on microbiota.

**Table 1:** ANOSIM R values and statistical significance of pairwise weighted Unifrac distance between microbiota collected at six timepoints

Comparison^a^	R	p
T0-T1	0.26	0.023
T0-T2	0.54	0.001^b^
T0-T3	0.08	0.217
T0-T4	0.25	0.039
T0-T5	0.25	0.001^b^
T1-T2	0.06	0.262^c^
T1-T3	−0.04	0.624
T1-T4	−0.04	0.579
T1-T5	0	0.375
T2-T3	0.15	0.138
T2-T4	−0.06	0.726
T2-T5	0.01	0.417
T3-T4	0.01	0.345^c^
T3-T5	0.12	0.094
T4-T5	−0.05	0.694

^a^ timepoint abbreviations as described in Materials and Methods

^b^ statistically significant R after correction for multiple comparisons

^c^ comparisons of samples collected immediately before and after ciclosporin and prednisone treatment

**Fig. 2:**
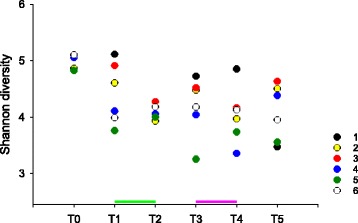
Microbiome diversity over time. Mean Shannon diversity for each animal is represented for each collection date. The means were calculated for samples collected from each dog at each timepoint (*n* = 2–6). Times of collection are represented on the x axis as defined in Materials and Methods. Dogs are colored as shown in the key. Green and pink bars indicate ciclosporin and prednisone treatment, respectively

To investigate another possible effect of the tested drugs, we estimated the α-diversity of the bacterial populations in swabbed samples using the Shannon [28] and Berger-Parker diversity index [29]. Altogether, the skin bacterial diversity was not signifcantly impacted by ciclosporin and prednisone treatment (Fig. 2).

Shannon diversity indices ranged in value between 3.4 and 5.1 regardless of treatment. We used Kruskal-Wallis one-way ANOVA on ranks to test whether microbiota diversity collected on the various dates differed. The test returned a significant *P*-value *(P =* 0.02). As for ANOSIM described above, comparisons between sampling dates were not significantly different when comparing samples collected immediately before and immediately after each treatment. Similarly, the analogous analysis based on Berger-Parker α-diversity showed no significant difference between pre- and post-treatment samples. In contrast to the analysis of Shannon diversity, no overall difference between sampling dates was found using one-way ANOVA (F_5,30_ = 2.205, *p* = 0.08). Together, Unifrac distances and diversity values indicate that neither ciclosporin nor prednisone had a detectable impact on the skin bacterial microbiota.

**Fig. 3:**
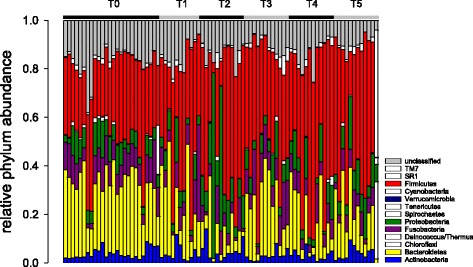
Phylum-level classification of bacterial microbiota from 85 skin swabs shows no apparent impact of treatment. Each bar represents a different sample. The most abundant phyla are colored as shown in the key. Timepoints are indicated uppermost. T1 and T2 indicate beginning and end of ciclosporin treatment, respectively; T3 and T4 indicate beginning and end of prednisone treatment, respectively. Within each timepoint (T), samples are ordered by dog (1 - > 6). The samples are from left to right: **T0**, 1_ing1, 1_ing2, 1_pinna1, 1_pinna2, 2_ing1, 2_ing2, 2_pinna1, 2_pinna2, 3_ing1, 3_ing2, 3_ing3, 3_pinna1, 3_pinna2, 3_pinna3, 4_ing1, 4_ing2, 4_pinna1, 4_pinna2, 5_ing1, 5_ing2, 5_pinna1, 5_pinna2, 6_ing1, 6_ing2, 6_pinna1, 6_pinna2. **T1**, 1_ing1, 1_pinna1, 2_ing1, 2_pinna1, 3_ing1, 3_pinna1, 4_ing1, 5_ing1, 5_pinna1, 6_ing1, 6_pinna1. **T2**, 1_ing1, 1_pinna1, 2_ing1, 2_pinna1, 3_ing1, 3_pinna1, 4_ing1, 4_pinna1, 5_ing1, 5_pinna1, 6_ing1, 6_pinna1. **T3**, 4_ing1, 4_pinna1, 5_ing1, 5_pinna1, 6_ing1, 6_pinna1, 1_ing1, 1_pinna1, 2_ing1, 2_pinna1, 3_ing1, 3_pinna1. **T4**, 1_ing1, 1_pinna1, 2_ing1, 2_pinna1, 3_ing1, 3_pinna1, 4_ing1, 4_pinna1, 5_ing1, 5_pinna1, 6_ing1, 6_pinna1. **T5**, 1_ing1, 1_pinna1, 2_ing1, 2_pinna1, 3_ing1, 3_pinna1, 4_ing1, 4_pinna1, 5_ing1, 5_pinna1, 6_ing1, 6_pinna1, where the number before the underscore indicates dog number and the final number within each code indicates left (1) and right (2) side. On timepoint T0 dog 3 was sampled three times, as indicated by (3)

The phylum-level classification of 85 swab samples is shown in Fig. 3. Consistent with PCoAs, neither ciclosporin nor prednisone induced any apparent change in microbiota taxonomy. A lower-level taxonomic classification shows the same lack of treatment effect (Additional files 2 and 3). Figure 3 shows a temporal trend of increasing relative abundance of Firmicutes over time. A linear regression of relative Firmicutes abundance over time was highly significant (F_1,23_ = 20.8, *P* < 0.001), but this trend appears to be unrelated to the tested interventions.

#### Diversity of skin microbiota between dogs and anatomical location

We analyzed 16S sequences to explore whether skin microbiota differed by dogs. Since all animals where subjected to the same treatment, this analysis is not central to the topic investigated here, but is relevant to understanding the evolution of skin microbiota and the extent to which co-housed animals may harbor different microbiota. Clustering by dog was found to be statistically significant (ANOSIM, *n* = 6, R = 0.25, *P* < 0.0001). Variation Partitioning Analysis was used to further evaluate the relative contribution of two independent variables, dog and time, on the microbiome profile. The results of this analysis show that variable “dog” explains 27.5% of the explained variation, which is 10 times more than the variation explained by variable “time” (2.6%). This outcome is consistent with the PCoA and ANOSIM results described above and in Additional file 4 in showing that time of sample collection had a relatively small impact on the skin microbiome.

When the samples from six dogs were separated by anatomical site, clustering of skin microbiota by dog became apparent by PCoA (Additional file 4) and was statistically significant when all dogs were compared (abdomen, *n* = 6, ANOSIM R = 0.40, *P* < 0.0001; pinna n = 6 ANOSIM R = 0.24, *P* < 0.001). After Bonferroni correction, 8/15 pairwise comparisons of abdomen microbiota were significantly different. For the pinna, only two out of 15 pairwise comparisons between dogs were significantly different (Additional file 5). α and β diversity was analyzed in relation to body site and to compare intra-dog vs inter-dog diversity. Based on Shannon diversity, samples collected from the abdomen and from the pinna were equally diverse (*n* = 93, *t* test *P* = 0.079). In contrast, samples collected from the same dog, whether from the pinna or the abdomen, were less different from each other than samples from different dogs (Kruskal-Wallis ANOVA on ranks, *P* < 0.001). This analysis indicates that in spite of the animals being housed together, the skin microbiota populating different animals tends to diverge, and that each dog is populated by a distinct bacterial microbiota.

### Quantification of *Malassezia* and association with bacterial diversity

A nested real-time PCR assay was used to quantify *Malassezia* DNA in the same swab samples as used for 16S rRNA sequencing. *Malassezia* carriage varied among dogs. At time T0, before initiation of treatment, some dogs had high *Malassezia* loads (i.e., dog 5) (Additional file 6). In contrast, other dogs showed very low loads (dogs 3, 6). Despite this trend, high inter-sample variability was observed among samples from a same dog. The density of *Malassezia* was higher on the pinnae than on the abdomen (Mann-Whitney Rank sum test, U = 2016, *n* = 72, *P* = 0.021). *Malassezia* populations significantly varied in the course of the experiment (ANOVA on Ranks, *n* = 24/group, H = 20.1, 5 d.f., *P* < 0.001). However, such variation did not appear to be a result of treatment (Additional file 6). Neither of the two pairwise comparisons between C_t_ values from samples taken immediately before and after the treatment were significantly different (Tukey’s test).

We analyzed *Malassezia* PCR C_t_ values and 16S rRNA diversity for an association between *Malassezia* infestation and bacterial diversity. For this analysis, we used both Shannon and Berger-Parker diversity indices. Bacterial diversity and *Malassezia* relative abundance were not significantly correlated (Additional file 7). Although none of the regressions were statistically significant, we observed a slight yet non-significant increase in bacterial diversity with increasing *Malassezia* C_t_ (decreasing *Malassezia* relative abundance) for the abdominal samples and an opposite trend for those collected on the pinnae.

### Detection of *Papillomavirus* DNA

The results of PCR to detect *Papillomavirus* DNA in the skin samples are summarized in Table 2; these results are in agreement with those from previous studies that had demonstrated that *Papillomavirus* DNA is detectable on the skin of healthy dogs. Dogs 2 and 4 were positive once and dog 3 was positive on two consecutive sampling points. As observed for the bacterial microbiota and *Malassezia* infestation, the results did not correlate with the treatments administered.

**Table 2:** Results of Papillomavirus PCR

Dog number and sampling site	Timepoint					
	T0	T1	T2	T3	T4	T5
1 Pinna	**–**	**–**	**–**	**–**	**–**	**–**
1 Abdomen	**–**	**–**	**–**	**–**	**–**	**–**
2 Pinna	**–**	**–**	**–**	**–**	**+**	**–**
2 Abdomen	**–**	**–**	**–**	**–**	**–**	**–**
3 Pinna	**–**	**+**	**+**	**–**	**–**	**–**
3 Abdomen	**–**	**–**	**–**	**–**	**–**	**–**
4 Pinna	**–**	**–**	**–**	**–**	**–**	**–**
4 Abdomen	**–**	**–**	**–**	**+**	**–**	**–**
5 Pinna	**–**	**–**	**–**	**–**	**–**	**–**
5 Abdomen	**–**	**–**	**–**	**–**	**–**	**–**
6 Pinna	**–**	**–**	**–**	**–**	**–**	**–**
6 Abdomen	**–**	**–**	**–**	**–**	**–**	**–**

## Discussion

The cutaneous microbiota of six atopic dogs was analyzed at six timepoints over a 7-months period. This study is unique because of its comprehensive examination of microbiota, including bacteria, *Malassezia and Papillomavirus.* To our knowledge, this is the first study that investigated the impact of non-antimicrobial treatments on the canine cutaneous microbiome. The major limitation of this study is the small number of dogs investigated. However, as we found no apparent trend when pre- and post-treatment samples were compared, it is unlikely that a study with more animals would lead to a different conclusion.

Our original hypothesis was that ciclosporin or glucocorticoids would reduce bacterial diversity, facilitating overgrowth of pathogens and the development of overt skin infections. Our results show that the treatment with ciclosporin at 5 mg/kg/q 24 h or with prednisone at 0.75 mg/kg/q 24 h does not impact the profile or the diversity of the cutaneous bacterial microbiome of the dog. Therefore, it is unlikely that treatment itself directly increases the risk of secondary bacterial infections commonly observed in atopic dogs. The specific trigger(s) of *Staphylococcus pseudintermedius* proliferation ending in pyoderma in an atopic dog remain unclear, but the leading hypothesis is that abnormalities in the skin barrier or immunologic changes linked to atopic dermatitis [30] lead to a reduction in bacterial diversity and facilitate bacterial infections, as reported in humans [10] and more recently in dogs [11]. In this study, we did not challenge dogs with the allergen to which they are sensitized, because the main objective was to assess the impact of the treatment - and not allergic inflammation - on the bacterial microbiota, on *Malassezia* and on *Papillomavirus* infection. The assessment of the bacterial microbiome after an allergen challenge in this acute canine atopic dermatitis model has been reported previously [31].

The detection and relative quantification of *Malassezia* DNA was successful using the PCR primers reported by Makimura et al. [27] which are specific for *Malassezia pachydermatis.* A nested PCR protocol was used to increase the sensitivity of this test. None of the two treatments had an impact on the relative abundance of *Malassezia* yeast and thus we concluded that ciclosporin and prednisolone do not affect cutaneous *Malassezia* populations under the conditions tested. The outbreaks of *M. pachydermatis* frequently observed in atopic dogs probably should be better attributed to changes in the cutaneous ecosystem caused by the atopic disease rather than to treatment itself. Previous studies have reported that in human atopic patients different *Malassezia* species are present on the skin, which is typically not the case in healthy individuals [32, 33]. Importantly, in this study, we only investigated the most common and clinically important *Malassezia* species in the dog (*M. pachydermatis*) and therefore cannot draw conclusions on the diversity of the entire *Malassezia* population, as reported in atopic humans [34]. A recent study based on high-throughput sequence data of the ribosomal internal transcribed spacer I analyzed the fungal population of the skin in healthy and atopic dogs [35]. This study found that the relative abundance of *Malassezia* was not impacted by the dog’s health status. This observation and our study are however difficult to compare. While Meason-Smith et al. used high-throughput sequencing to infer relative abundance, we quantified *Malassezia* using real-time PCR. In contrast to the earlier study, our experiments were not designed to assess differences in skin microbial populations between atopic and healthy animals.

As for the analysis of bacterial microbiota and *Malassezia*, we found that the treatments did not increase the presence of detectable *Papillomavirus* in the skin. As expected, papillomavirus DNA was detected in some dogs at different points of the study (Table 2), but all dogs were negative for this group of commensals at the end of the study.

## Conclusions

Ciclosporin and prednisone, used at typical dosages prescribed for the treatment of atopic dermatitis for one month did not impact the canine cutaneous microbiota in a detectable manner. The data suggest that these treatments are unlikely to increase the risk of secondary microbial infections commonly observed in atopic dogs. Whether a longer treatment would change the skin microbiota remains undetermined but deserving of further study.

## Additional files

Additional file 1: Sample replication and experimental noise. Three samples were replicated to estimate the level of technical variation. Replicated samples are shown in black. For clarity some black data points were slightly shifted on the plot to eliminate symbol overlap. The distance between replicates represents the experimental noise caused by PCR and sequencing. The mean weighted Unifrac distance among 4275 non-replicated pairwise comparisons (93 × 92/2–3 = 4275) was 0.383, whereas the mean for the three pairwise distance between replicates was 0.186. Unifrac distance values originating from experimental noise was thus significantly smaller than the average Unifrac distance from different samples (Mann-Whitney Rank Sum Test U = 456, *p* = 0.005). Triangle up, dog 1 abdomen; triangle down, dog 2 pinna; diamond, dog 3 abdomen. The percent variation explained by the first two principal axes is indicated. (DOCX 16 kb)

Additional file 2: Example of lower-level classification of skin microbiota over time for dog 1 and dog 3. Bars are arranged from left to right in chronological order grouped by anatomical site. Stacks are arranged from bottom to top in order of diminishing cumulative taxon abundance across all samples. The 20 most abundant taxa are shown. Bars height is < 1 because less abundant taxa are not shown. Sequences classified at the genus level with 70% probability value were assigned to the next higher taxonomic level i.e., family, order, class or phylum. The dates when the samples were collected are abbreviated as DDMMYY. On 5/21/14 (T1), dog 1 samples were collected in duplicate and dog 3 samples in triplicate. Replicate samples were barcoded individually to visualize experimental variation (see Additional file 1). The dogs showed a different composition of the microbiome, which remained relatively constant over time. As seen in the other analyses, no effect of treatment on microbiota composition was apparent. (DOCX 265 kb)

Additional file 3: Genus-level classification of abdomen and pinna skin microbiota from six dogs. (DOCX 38 kb)

Additional file 4: Principal Coordinates Analysis by body site. When two sampling locations are considered separately, clustering by dog is significant (pinna *n* = 6, ANOSIM R = 0.24, *P* < 0.001; abdomen, n = 6, ANOSIM R = 0.40, *P* < 0.0001). The data points are color-coded by dog as shown in Fig. S1. A, pinna; B, abdomen. (DOCX 34 kb)

Additional file 5: Pairwise ANOSIM R values and statistical significance level for comparisons of weighted Unifrac distance between microbiota from six dogs and two body sites. (DOCX 13 kb)

Additional file 6: Temporal evolution of *Malassezia* infestation of pinna and inguinal area of six dogs. The timepoints are labelled as indicated in Materials and Methods. Data points are colored according to dog as in Fig. 1. Swabs from the left and right side were analyzed individually as shown by duplicated lines of same color. Ciclosporin and prednisone treatment are represented with a green and a pink bar, respectively. Crossing points exceeding 41 cycles (< 10 copies) were deemed negative and are not represented in graphs. Triangles indicate positive control. Crossing points were converted to number of ITS1 copies using the standard curve. (DOCX 31 kb)

Additional file 7: Lack of significant association between *Malassezia* abundance and bacterial diversity. Left and right graph show the analysis of inguinal and pinna samples, respectively. Full symbols indicate Shannon diversity, empty symbols Berger-Parker diversity. Linear regression model is indicated by lines. (DOCX 43 kb)

## Abbreviations

ANOVA: Analysis of Variance
nt: nucleotide
OTU: Operational Taxonomic Unit
PCoA: Principal Coordinates Analysis
rRNA: ribosomal RNA
SD: standard deviation
