# Supplementary figures and images for: Glucocorticosteroids and ciclosporin do not significantly impact canine cutaneous microbiota
Source: BMC Vet Res. 2018 Feb 23;14:51. doi: 10.1186/s12917-018-1370-y (PMC5824610; doi:10.1186/s12917-018-1370-y)

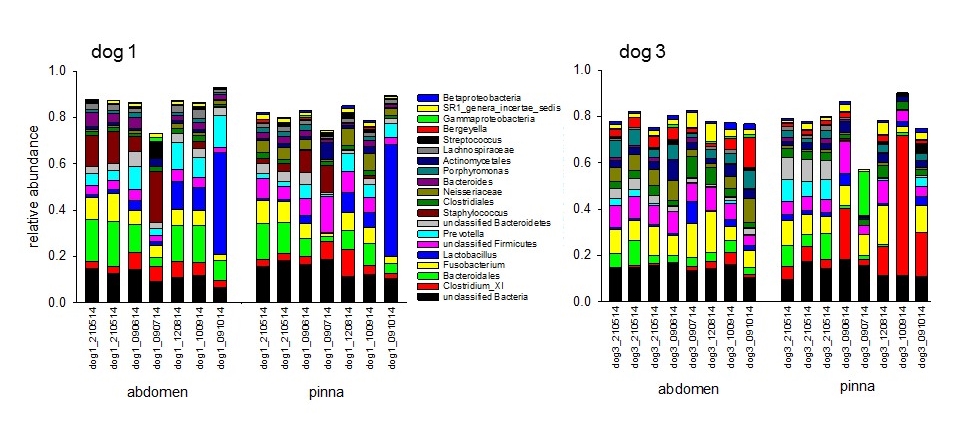

Supplement: Supplementary file 2 — Example of lower-level classification of skin microbiota over time for dog 1 and dog 3. Bars are arranged from left to right in chronological order grouped by anatomical site. Stacks are arranged from bottom to top in order of diminishing cumulative taxon abundance across all samples. The 20 most abundant taxa are shown. Bars height is < 1 because less abundant taxa are not shown. Sequences classified at the genus level with 70% probability value were assigned to the next higher taxonomic level i.e., family, order, class or phylum. The dates when the samples were collected are abbreviated as DDMMYY. On 5/21/14 (T1), dog 1 samples were collected in duplicate and dog 3 samples in triplicate. Replicate samples were barcoded individually to visualize experimental variation (see Additional file 1). The dogs showed a different composition of the microbiome, which remained relatively constant over time. As seen in the other analyses, no effect of treatment on microbiota composition was apparent. (DOCX 265 kb) [file 12917_2018_1370_MOESM2_ESM.docx]

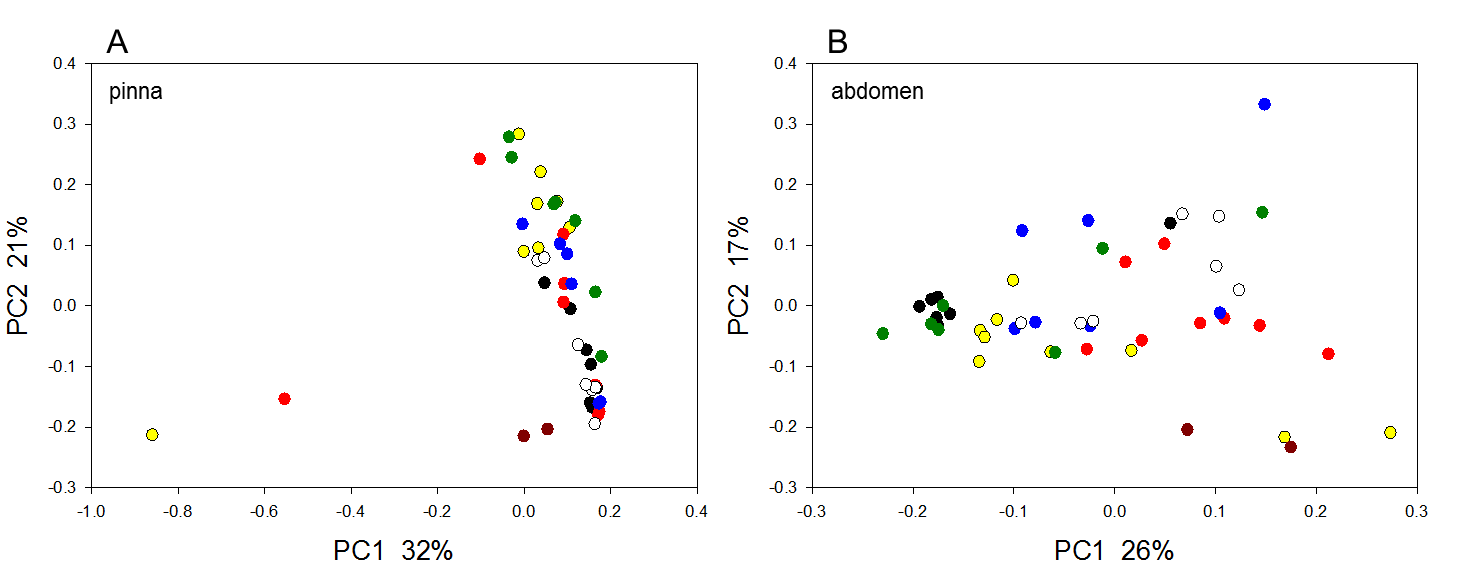

Supplement: Supplementary file 4 — Principal Coordinates Analysis by body site. When two sampling locations are considered separately, clustering by dog is significant (pinna n = 6, ANOSIM R = 0.24, P < 0.001; abdomen, n = 6, ANOSIM R = 0.40, P < 0.0001). The data points are color-coded by dog as shown in Fig. S1. A, pinna; B, abdomen. (DOCX 34 kb) [file 12917_2018_1370_MOESM4_ESM.docx]

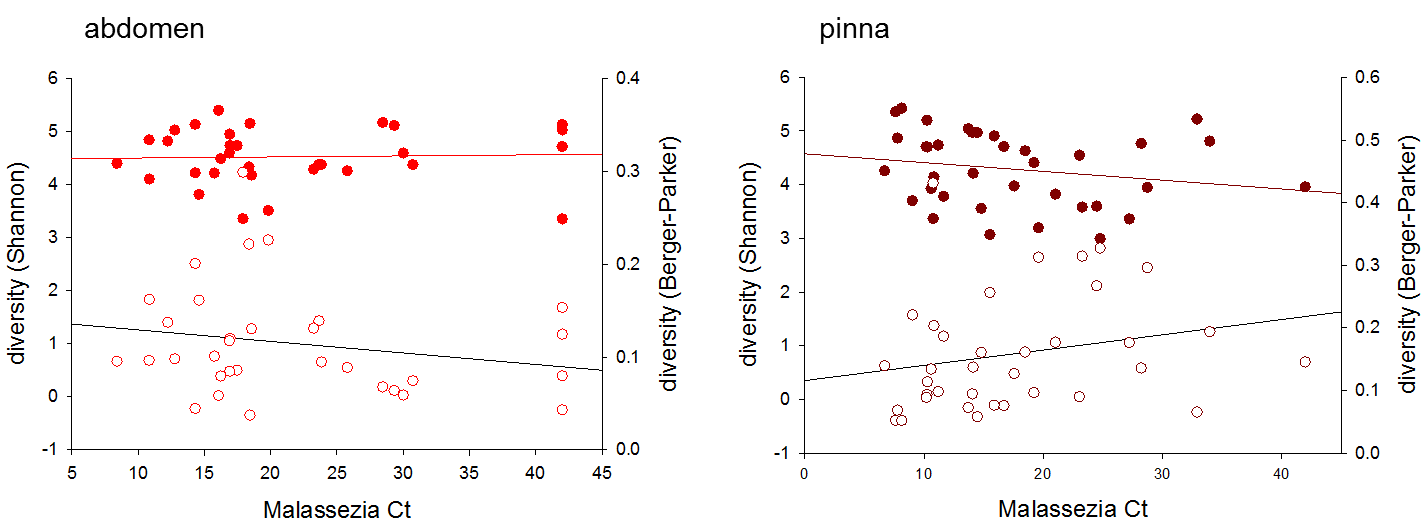

Supplement: Supplementary file 7 — Lack of significant association between Malassezia abundance and bacterial diversity. Left and right graph show the analysis of inguinal and pinna samples, respectively. Full symbols indicate Shannon diversity, empty symbols Berger-Parker diversity. Linear regression model is indicated by lines. (DOCX 43 kb) [file 12917_2018_1370_MOESM7_ESM.docx]
