# Supplementary material for: Glucocorticosteroids and ciclosporin do not significantly impact canine cutaneous microbiota
Source: BMC Vet Res. 2018 Feb 23;14:51. doi: 10.1186/s12917-018-1370-y (PMC5824610; doi:10.1186/s12917-018-1370-y)
Supplement: Supplementary file 3 — Genus-level classification of abdomen and pinna skin microbiota from six dogs. (DOCX 38 kb) [file 12917_2018_1370_MOESM3_ESM.docx]

Genus-level classification of abdomen and pinna skin microbiota from six dogs^a^.

^a^ 20 most abundant genera are shown
