# Supplementary material for: Glucocorticosteroids and ciclosporin do not significantly impact canine cutaneous microbiota
Source: BMC Vet Res. 2018 Feb 23;14:51. doi: 10.1186/s12917-018-1370-y (PMC5824610; doi:10.1186/s12917-018-1370-y)
Supplement: Supplementary file 5 — Pairwise ANOSIM R values and statistical significance level for comparisons of weighted Unifrac distance between microbiota from six dogs and two body sites. (DOCX 13 kb) [file 12917_2018_1370_MOESM5_ESM.docx]

**Pairwise ANOSIM R values and statistical significance level for pairwise comparions of weighted Unifrac distance between microbiota from six dogs and two body sites.**

| comparison | Abdomen |  | Pinna |  |
| --- | --- | --- | --- | --- |
|  | R | p | R | p |
| 1 - 2 | 0.24 | 0.0022^a^ | 0.53 | 0.0004^a^ |
| 1 - 3 | 0.77 | 0.0001^a^ | 0.07 | 0.1044 |
| 1 - 4 | 0.28 | 0.0030 | 0.16 | 0.0512 |
| 1 - 5 | 0.00 | 0.3845 | 0.52 | 0.0029 |
| 1 - 6 | 0.62 | 0.0011^a^ | 0.15 | 0.0456 |
| 2 - 3 | 0.54 | 0.0008^a^ | 0.27 | 0.0050 |
| 2 - 4 | 0.26 | 0.0045 | 0.22 | 0.0277 |
| 2 - 5 | 0.16 | 0.0242 | 0.24 | 0.0066 |
| 2 - 6 | 0.59 | 0.0002^a^ | 0.52 | 0.0007^a^ |
| 3 - 4 | 0.50 | 0.0001^a^ | 0.01 | 0.3869 |
| 3 - 5 | 0.65 | <0.0001^a^ | 0.30 | 0.0050 |
| 3 - 6 | 0.55 | 0.0004^a^ | 0.09 | 0.1181 |
| 4 - 5 | 0.10 | 0.1057 | 0.17 | 0.0935 |
| 4 - 6 | 0.28 | 0.0093 | 0.04 | 0.2737 |
| 5 - 6 | 0.49 | 0.0024 | 0.32 | 0.0160 |

^a^ statistically significant R after correction for multiple comparisons
