# Supplementary material for: Glucocorticosteroids and ciclosporin do not significantly impact canine cutaneous microbiota
Source: BMC Vet Res. 2018 Feb 23;14:51. doi: 10.1186/s12917-018-1370-y (PMC5824610; doi:10.1186/s12917-018-1370-y)
Supplement: Supplementary file 4 [file 12917_2018_1370_MOESM1_ESM.docx]

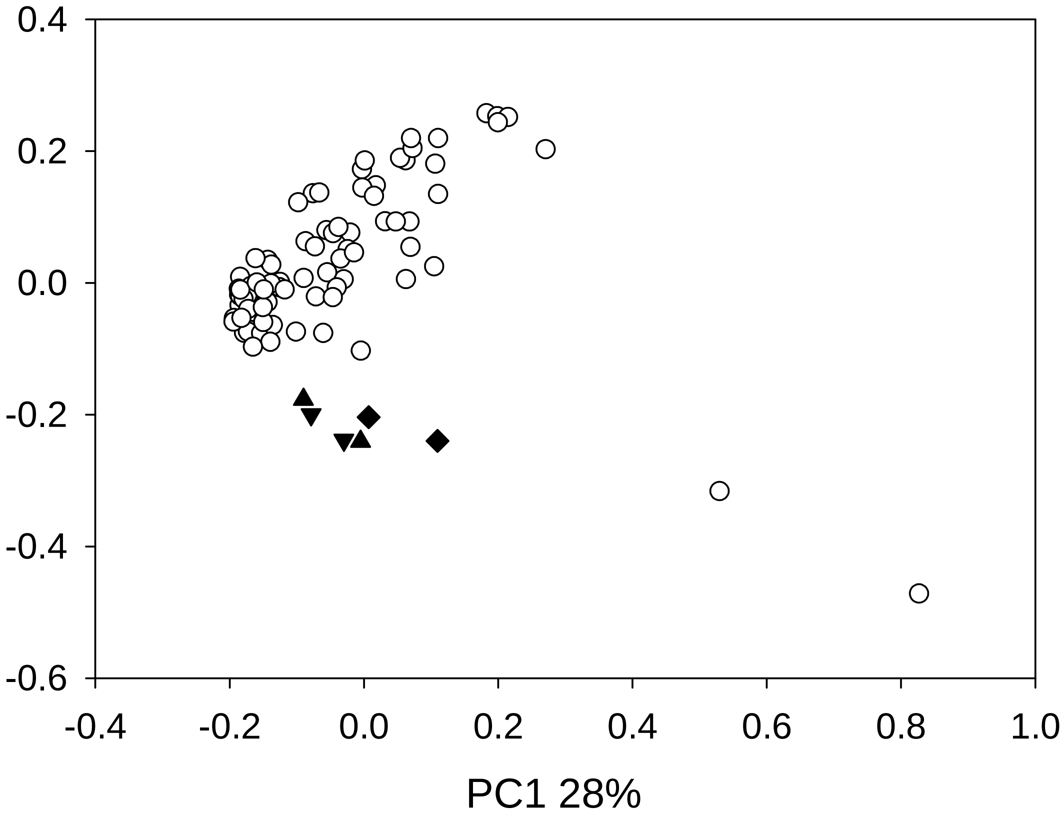
0.4
0.2
0.0
-0.2
-0.4
-0.6
-0.4
-0.2
0.0
0.2
0.4
0.6
0.8
1.0
PC1 28%
