# Supplementary material for: Glucocorticosteroids and ciclosporin do not significantly impact canine cutaneous microbiota
Source: BMC Vet Res. 2018 Feb 23;14:51. doi: 10.1186/s12917-018-1370-y (PMC5824610; doi:10.1186/s12917-018-1370-y)
Supplement: Supplementary file 5 [file 12917_2018_1370_MOESM6_ESM.docx]

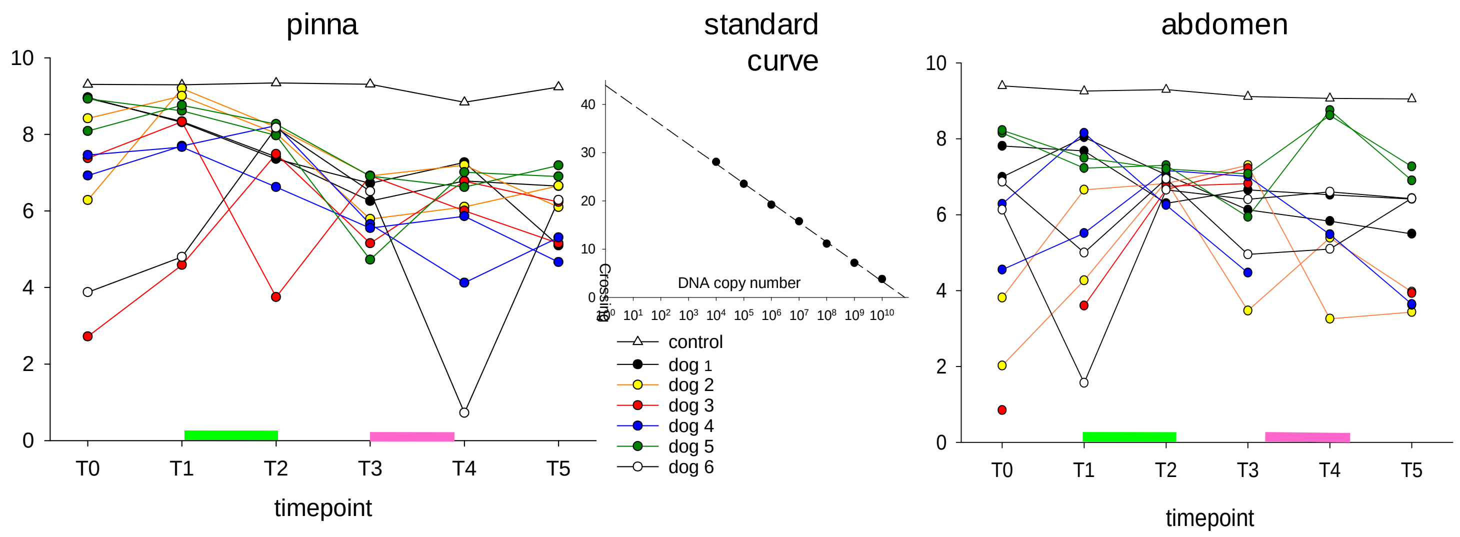

pinna
10
8
6
4
2
0
T0
T1
T2
T3
T4
T5
timepoint
standard curve
40
30
20
10
0
DNA copy number
control
dog 1
dog 2
dog 3
dog 4
dog 5
dog 6
abdomen
timepoint
